# Association between hemoglobin-to-red cell distribution width ratio and depression in Chinese adults: a population-based cross-sectional study

**DOI:** 10.3389/fpubh.2025.1638290

**Published:** 2025-09-16

**Authors:** Yike Xu, Shuwen Zhang, Yang Liu, Junteng Zhou

**Affiliations:** ^1^West China School of Medicine, West China Hospital, Sichuan University, Chengdu, China; ^2^Medical Imaging Key Laboratory of Sichuan Province, North Sichuan Medical College, Nanchong, Sichuan, China; ^3^Department of Cardiology and Laboratory of Cardiovascular Diseases, West China Hospital, Institute of Cardiovascular Diseases, Sichuan University, Chengdu, Sichuan, China; ^4^Division of Vascular Surgery, Department of General Surgery, and Laboratory of Cardiovascular Diseases, West China Hospital, Sichuan University, Chengdu, China; ^5^Health Management Center, General Practice Medical Center, West China Hospital, Sichuan University, Chengdu, China

**Keywords:** hemoglobin-to-red cell distribution width ratio, depression, graded statistical association, Chinese population, cross-sectional study

## Abstract

**Objective:**

This study investigates the hemoglobin-to-red blood cell distribution width ratio (HRR) and its association with risk of elevated depressive symptoms in Chinese adults, addressing a gap in evidence for non-older populations and exploring potential effect modifiers.

**Methods:**

In this cross-sectional analysis of 30,427 adults from routine health screenings (July 2020–June 2021), depression was assessed using the Self-Rating Depression Scale. HRR was calculated as hemoglobin (g/dL) divided by red cell distribution width (%). Multivariable logistic regression, restricted cubic splines (RCS), sensitivity analyses with alternative depression definitions, E-value analysis, and ROC curve comparisons with the RDW-to-albumin ratio (RAR) were conducted, adjusted for sociodemographic, lifestyle, and clinical confounders.

**Results:**

In a population of 30,427 adults (46.3% female; mean age 44.9 ± 10.7 years; 56.7% with college education or above) undergoing routine health screenings, a strong inverse graded statistical association was observed between hemoglobin-to-red cell distribution width ratio (HRR) and elevated depressive symptoms (SDS ≥ 53). Each unit increase in HRR reduced elevated depressive symptoms (SDS ≥ 53) by 39% in fully adjusted models (OR = 0.61, 95% CI: 0.51–0.72, *p* < 0.0001). After adjusting covariates, compared to the lowest quartile (Q1: HRR ≤ 1.031), participants in Q2 (1.031–1.142), Q3 (1.142–1.25), and Q4 (≥1.25) exhibited 10% (OR = 0.90, 95% CI: 0.84–0.98), 13% (OR = 0.87, 95% CI: 0.80–0.94), and 24% (OR = 0.76, 95% CI: 0.70–0.83,) reductions in elevated depressive symptoms (SDS ≥ 53), respectively (*p*-trend<0.0001). RCS confirmed a linear association without threshold effects. Age modified the relationship (*p*-interactio*n* = 0.004), with stronger protection in adults ≥45 years (OR = 0.40, 95% CI: 0.32–0.51) than younger individuals (OR = 0.64, 95% CI: 0.50–0.82).

**Conclusion:**

HRR may indicate an independent, linear inverse association with elevated depressive symptoms (SDS ≥ 53) in Chinese adults, with strong effects in older populations. As a cost-effective hematological biomarker, HRR could support scalable elevated depressive symptoms (SDS ≥ 53) stratification and prevention strategies to complement other risk factors for elevated depressive symptoms, particularly in aging groups, and these findings warrant validation in prospective studies.

## Introduction

Depression is a globally prevalent mental health disorder and a leading contributor to disability and disease burden (1). According to the World Health Organization, more than 280 million individuals suffer from depression worldwide, with growing prevalence in low- and middle-income countries (2). In China, approximately 17% of adults experience depressive symptoms during their lifetime, posing significant challenges to public health systems (3). While the etiology of depression is multifactorial—including genetic, psychological, and environmental influences—emerging evidence highlights the role of systemic inflammation and oxidative stress in its pathophysiology (4). Against this backdrop, routinely available hematological indicators, such as hemoglobin (Hb) (5) and red cell distribution width (RDW), have gained research interest due to their potential to reflect inflammatory and metabolic states relevant to mental health. Prior studies have shown that lower Hb and elevated RDW levels are each associated with increased depressive symptoms. Elevated red cell distribution width (RDW) reflects inflammation and oxidative stress-driven erythrocyte size heterogeneity, directly implicating pathways associated with depression pathogenesis (6, 7). Concurrently, reduced hemoglobin (Hb) compromises oxygen delivery to neural tissues, potentially disrupting cerebral energy metabolism and monoamine neurotransmitter synthesis (e.g., serotonin, dopamine) (8).

Recently, attention has shifted to composite biomarkers such as the hemoglobin-to-red cell distribution width ratio (HRR), which may offer a more integrated reflection of physiological changes linked to depression. The hemoglobin-to-RDW ratio (HRR) integrates these dual dimensions—oxygen transport capacity and erythrocyte heterogeneity—thereby capturing synergistic physiological stressors (inflammatory, hypoxic) more comprehensively than either marker alone (9). Critically, HRR’s derivation from routine complete blood counts (CBC) offers significant clinical advantages: low cost (<$5/test), immediate availability in standard laboratories, and scalability for primary care/community depression screening programs. We note that both hemoglobin and red cell distribution width are influenced by multiple clinical and environmental factors — including nutritional deficits (e.g., iron, folate, vitamin B12), renal dysfunction, chronic inflammatory states, and medication exposures — many of which also co-vary with depressive symptoms. Consequently, HRR should be interpreted as an indirect, integrative marker that may reflect these overlapping physiological processes rather than a specific causal factor for depression. Given the multifactorial nature of depression and the complex interplay between hematological parameters and various health conditions, substantial potential for residual confounding from unmeasured factors (such as detailed nutritional status, inflammatory markers, medication effects, and psychosocial stressors) must be acknowledged. This study therefore aims to explore HRR as a hypothesis-generating proxy indicator while recognizing these inherent limitations in observational research.

A large cross-sectional study focusing on older adults (10) reported a significant inverse association between HRR and depression; however, evidence remains limited regarding younger and more diverse adult populations. To address this gap, the present study utilizes data from the Health Management Center of West China Hospital (11), a large-scale clinical database comprising general adult individuals undergoing routine health examinations. This population-based approach enables a comprehensive assessment of the relationship between HRR and risk of elevated depressive symptoms beyond the older adults, while also exploring potential modifying factors such as age, lifestyle, and comorbid conditions. Therefore, this cross-sectional study aimed to investigate the association between HRR and depression in a large Chinese adult population. By addressing these aims, we hope to advance the use of HRR in scalable mental health risk assessment frameworks.

## Methods

### Study population

**Figure 1:**
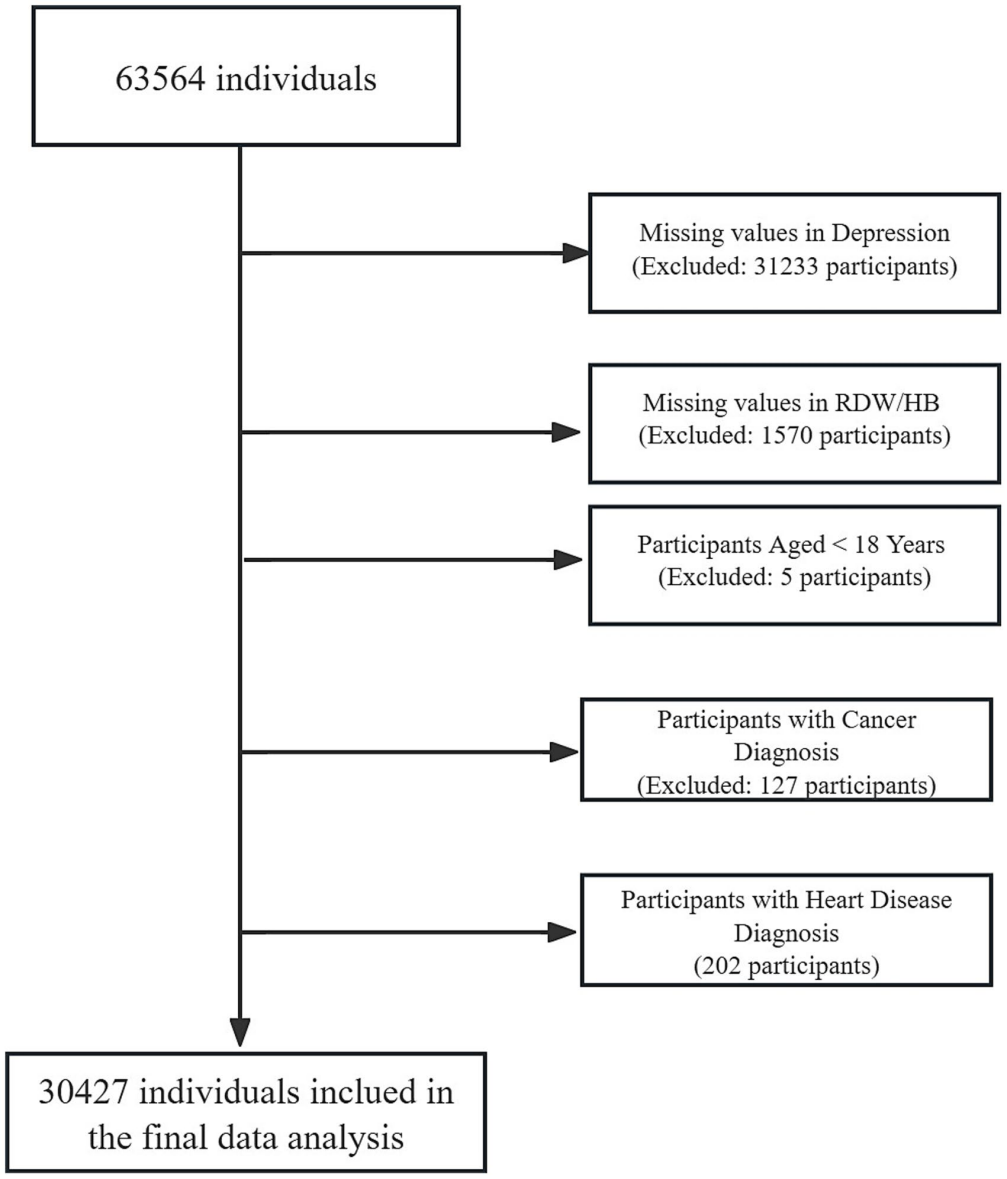
Flowchart of participant inclusion and exclusion criteria.

As previously reported, from July 2020 to June 2021, 63,564 adults completed routine health screenings at West China Hospital’s Health Management Center (11). After applying exclusion criteria: 49.0% (*n* = 31,233) were removed due to incomplete depression assessments, followed by 5% (*n* = 1,570) with missing hematological biomarkers (RDW/HB). Subsequent exclusions comprised age <18 years (*n* = 5), cancer diagnoses (*n* = 127), and cardiovascular conditions (*n* = 202), resulting in 30,427 eligible participants (Figure 1). Specifically, cardiovascular disease (CVD) and cancer were excluded because these conditions can substantially alter hematologic indices (including HRR) and risk of elevated depressive symptoms through systemic inflammation, metabolic disturbances, treatment effects, and comorbidity burden. Furthermore, they introduce complex bidirectional relationships (e.g., depression as a risk factor for CVD and a prognostic factor in cancer) that obscure the independent association between HRR and depression (12, 13). Comparative analysis revealed no meaningful differences in baseline characteristics between the excluded individuals and the final group (Supplementary Table S1).

This investigation analyzed a single-institution clinical dataset from the Health Management Center of West China Hospital (Chengdu, China) collected during in-person health examinations; it was not a secondary analysis of NHANES or any other open repository. All inclusion/exclusion criteria (including *a priori* exclusion of cardiovascular disease and cancer due to strong bidirectional confounding on hematological indices and depression) were prespecified before analysis, and the participant flow and comparability of excluded vs. included samples are documented. The Ethics Committee of West China Hospital approved this research (No. 2018–303), with all participants providing written consent.

### Depressive symptoms assessment

Depressive symptoms were evaluated using the Chinese version of the Zung Self-Rating Depression Scale (SDS), originally developed by Zung in 1965 (14). The SDS, a 20-item validated instrument, was administered to assess depressive symptoms experienced over the preceding 7 days. Each item employs four frequency-based responses: (1) never, (2) sometimes, (3) frequently, and (4) always. The raw score (sum of all items, range 20–80) was converted to a standardized score by multiplying by 1.25. In this study, we implemented a threshold of 53 for depression screening (standardized score ≥53 indicating clinically significant symptoms) (15). The Zung Self-Rating Depression Scale (SDS) was administered by trained clinicians using standardized protocols, with a cutoff of ≥53 selected based on validation studies in Chinese populations demonstrating robust sensitivity/specificity (16). While differing cutoffs exist across settings, this threshold aligns with our center’s standardized practice calibrated against clinical evaluations. Questionnaires were completed during routine health examinations. However, while SDS has acceptable psychometric properties in Chinese populations, it is a screening tool rather than a diagnostic interview; therefore it provides information on symptom burden rather than a clinical DSM/ICD diagnosis established by structured interview. In this study we operationalized the outcome as “elevated depressive symptoms (SDS ≥ 53),” consistent with our center’s screening practices and published validation studies. To mitigate administration variability, participants self-administered the SDS within 20–30 min under standardized conditions; for individuals with visual impairments or limited literacy (<6 years of education), trained staff neutrally read items without interpretive guidance to ensure unbiased responses.

### Measurement of HRR and covariates

After fasting overnight for 10–12 h, peripheral blood samples were collected in the morning by experienced nurses at the Health Management Center of West China Hospital using EDTA anticoagulant tubes. Hematological parameters including hemoglobin (Hb) and red blood cell distribution width (RDW) were analyzed following standardized laboratory protocols (17). The hemoglobin-to-red blood cell distribution width ratio (HRR) was calculated as hemoglobin (g/dL) ÷ RDW (%) measured by automated hematology analyzers, and for clarity, HRR values were uniformly reported with two decimal places throughout the manuscript. Covariates encompassed demographic factors age, sex, occupation and education level, lifestyle indicators (smoking status and alcohol use), clinical parameters (hypertension, diabetes, hyperlipidemia, kidney disease), and anthropometric measures (BMI). It is worth mentioning that, to improve precision of confounding control, we specifically included kidney disease as an additional covariate in our analysis. Kidney disease status was determined based on participants’ self-report of having ever been told by a health professional that they had kidney disease, consistent with prior epidemiological studies.

Covariates were selected based on established risk factors for depression identified in previous literature and clinical relevance (18). Age and sex were included as fundamental demographic variables. BMI, smoking, and alcohol consumption represent modifiable lifestyle factors (19). Education and occupation reflect socioeconomic status (20). Hypertension and diabetes were included as they may share inflammatory pathways with depression (21). Data collection adhered to consistent methodologies and quality controls as in our prior work (11).

### Statistical analysis

In our study, analyses were prespecified around a single primary exposure (HRR) and a single primary outcome (SDS-defined depression). Multivariable models were used throughout; no univariate screening was employed to select covariates. Subgroup and ROC analyses were considered exploratory; exact *p*-values are reported and interpretation emphasizes effect sizes and confidence intervals rather than multiplicity-driven dichotomization. All the reporting follows the STROBE Statement (22). Continuous variables were presented as mean ± standard deviation (SD) and categorical variables as frequencies (percentages). To assess the normality of continuous variables, we use the Shapiro–Wilk test combined with visual inspection of Q-Q plots and histograms. Variables following normal distribution were presented as mean ± standard deviation, while non-normally distributed variables were presented as median (interquartile range). Between-group comparisons (depressed vs. non-depressed) utilized Student’s t-tests for continuous measures and χ^2^ tests for proportions.

Logistic regression models quantified associations between HRR (analyzed both continuously and by quartiles) and risk of elevated depressive symptoms across four sequential adjustments: crude model (unadjusted), Model 1 (age, sex), Model 2 (+BMI, smoking, alcohol use), and Model 3 (+education, occupation, chronic diseases). Covariates were adjusted across sequential models given their established dual associations with both depression pathophysiology and hematological regulation—where age, sex, lifestyle factors, and cardiometabolic conditions demonstrably influence HRR values while concurrently modifying risk of elevated depressive symptoms through shared inflammatory pathways. This hierarchical adjustment strategy specifically addresses the biological interdependence observed in our stratified analyses, ensuring HRR’s independent association is isolated from confounding by these covarying factors. In addition, we computed E-values for the point estimate and the 95% CI lower bound to evaluate the minimum strength of association an unmeasured confounder would need with both exposure and outcome to move the observed OR to the null (23). We emphasize that because exposure and outcome were measured concurrently, the study design is cross-sectional and precludes causal inference. The analyses thus quantify associations and should be treated as hypothesis-generating. We computed E-values for the point estimates and the lower bound of the 95% CI to quantify the minimum strength of association that an unmeasured confounder would need with both exposure and outcome to fully account for the observed association. While informative, E-values do not eliminate the possibility of residual confounding.

To address multicollinearity concerns arising from the mathematical relationship HRR = hemoglobin/RDW, we employed generalized variance inflation factor (GVIF) diagnostics. Crucially, regression models never simultaneously included HRR along with its component variables (hemoglobin or RDW) to avoid inherent mathematical collinearity. For all covariates, GVIF values were computed, with degrees-of-freedom adjusted values [GVIF^(1/(2*Df))] calculated for multi-categorical variables (e.g., education, occupation). Adjusted values exceeding √5 (≈2.24) were considered indicative of problematic collinearity (24).

Subgroup analyses assessed interaction effects through stratified models adjusted for covariates excluding the stratification variable, with heterogeneity tested via multiplicative interaction test. Analyses were conducted using R 4.2.3 and Free Statistics software (version 2.0; Beijing Free Clinical Medical Technology Co., Ltd., Beijing, China), with two-tailed *p* < 0.05 defining significance. Receiver operating characteristic (ROC) curve analysis was conducted to compare the discriminatory performance of HRR against conventional hematological markers (hemoglobin and RDW) for depression identification. Areas under the curve (AUCs) were calculated with 95% confidence intervals, and statistical comparisons between ROC curves were performed using DeLong’s test.

## Results

### Baseline characteristics of participants

The study population was carefully selected to balance scientific rigor with real-world relevance. We excluded conditions like heart disease and cancer since they independently affect both blood markers and risk of elevated depressive symptoms, while retaining anemia and kidney issues as these may naturally link to elevated depressive symptoms development. This approach was validated through sensitivity checks and population comparisons, with full documentation in Supplementary Materials. This study included 30,427 Chinese adults, with 6,261 (20.58%) exhibiting depressive symptoms (Table 1). Compared to non-depressed individuals, those with elevated depressive symptoms demonstrated distinct socio-demographic patterns: higher female proportion (53.62% vs. 44.38%), lower educational attainment (college or above: 42.17% vs. 60.50%), and overrepresentation in agricultural/industrial occupations (10.99% vs. 6.46%) (all *p* < 0.0001). Metabolic profiles revealed elevated diabetes prevalence in the elevated depressive symptoms group (9.25% vs. 7.59%, *p* < 0.0001), though hypertension and hyperlipidemia showed comparable rates. Hematological parameters exhibited significant divergence, with depressed participants showing lower hemoglobin (14.60 ± 1.71 vs. 14.82 ± 1.66 g/dL), higher RDW (13.23 ± 1.21% vs. 13.16 ± 1.14%), and consequently reduced HRR (1.11 ± 0.17 vs. 1.14 ± 0.17, all *p* < 0.0001). Lifestyle factors also showed differences, with current smoking slightly more prevalent in elevated depressive symptoms group (22.70% vs. 20.50%, *p* < 0.001).

**Table 1:** Baseline characteristics of participants by depression status.

Variables	Total (*n* = 30,427)	No (*n* = 24,166)	Yes (*n* = 6,261)	*p*-value
Age	44.91 ± 10.72	44.79 ± 10.56	45.38 ± 11.27	<0.001
Sex				<0.0001
Female	14,082 (46.28)	10,725 (44.38)	3,357 (53.62)	
Male	16,345 (53.72)	13,441 (55.62)	2,904 (46.38)	
BMI, kg/m2	23.73 ± 3.59	23.77 ± 3.61	23.58 ± 3.54	<0.001
Smoke				<0.001
Current	6,375 (20.95)	4,954 (20.50)	1,421 (22.70)	
Never	22,800 (74.93)	18,185 (75.25)	4,615 (73.71)	
Past	1,252 (4.11)	1,027 (4.25)	225 (3.59)	
Drink				<0.01
Current	3,435 (11.29)	2,792 (11.55)	643 (10.27)	
Never	26,754 (87.93)	21,195 (87.71)	5,559 (88.79)	
Past	238 (0.78)	179 (0.74)	59 (0.94)	
Occupation				<0.0001
Agriculture/Industrial	2,249(7.39)	1,561(6.46)	688 (10.99)	
Freelance/Other	11,079 (36.41)	8,587 (35.53)	2,492 (39.80)	
Government/Institution	12,466 (40.97)	10,474 (43.34)	1992 (31.82)	
Not record	1,602 (5.27)	1,244 (5.15)	358 (5.72)	
Student/Retired	3,031 (9.96)	2,300 (9.52)	731 (11.68)	
Education				<0.0001
College or above	17,261 (56.73)	14,621 (60.50)	2,640 (42.17)	
Elementary school or below	3,478 (11.43)	2,518 (10.42)	960 (15.33)	
Not record	1,569 (5.16)	1,216 (5.03)	353 (5.64)	
Secondary school or vocational school	8,119 (26.68)	5,811 (24.05)	2,308 (36.86)	
Hypertension				0.70
No	25,221 (82.89)	20,042 (82.93)	5,179 (82.72)	
Yes	5,206 (17.11)	4,124 (17.07)	1,082 (17.28)	
Diabetes				<0.0001
No	28,014 (92.07)	22,332 (92.41)	5,682 (90.75)	
Yes	2,413 (7.93)	1834 (7.59)	579 (9.25)	
Hyperlipidemia				0.92
No	29,933 (98.38)	23,775 (98.38)	6,158 (98.35)	
Yes	494 (1.62)	391 (1.62)	103 (1.65)	
Kidney disease				0.48
No	30,323 (99.66)	24,080 (99.64)	6,243 (99.71)	
Yes	104 (0.34)	86 (0.36)	18 (0.29)	
Hb (g/dL)	14.78 ± 1.67	14.82 ± 1.66	14.60 ± 1.71	<0.0001
RDW (%)	13.18 ± 1.15	13.16 ± 1.14	13.23 ± 1.21	<0.0001
HRR	1.13 ± 0.17	1.14 ± 0.17	1.11 ± 0.17	<0.0001

HRR, hemoglobin-to-red blood cell distribution width ratio.

To further examine the robustness of the HRR-depression association, we stratified participants into quartiles based on HRR values (Supplementary Table S2). The HRR quartiles were defined as Q1 (≤1.031, *n* = 7,559), Q2 (1.031–1.142, *n* = 7,663), Q3 (1.142–1.25, *n* = 7,705), and Q4 (≥1.25, *n* = 7,500). Quartile-based analysis revealed a graded inverse association between HRR and elevated depressive symptoms: compared with the lowest quartile (Q1 ≤ 1.031), participants in Q2 (1.031–1.142), Q3 (1.142–1.25), and Q4 (≥1.25) exhibited progressively lower odds of elevated depressive symptoms. The proportion of participants with elevated depressive symptoms decreased progressively across quartiles: 23.77% in Q1, 21.36% in Q2, 19.71% in Q3, and 17.44% in Q4 (*p* < 0.0001). This represents a reduction in elevated depressive symptoms prevalence from the lowest to highest HRR quartile. The quartile analysis also revealed systematic variations in demographic and clinical characteristics: higher HRR quartiles were associated with younger age, higher BMI, better educational attainment, increased prevalence of government/institutional employment, and higher rates of metabolic conditions including hypertension, diabetes, and hyperlipidemia. Notably, higher HRR quartiles differed systematically in these sociodemographic and clinical features.

### Association between HRR and risk of elevated depressive symptoms

**Figure 2:**
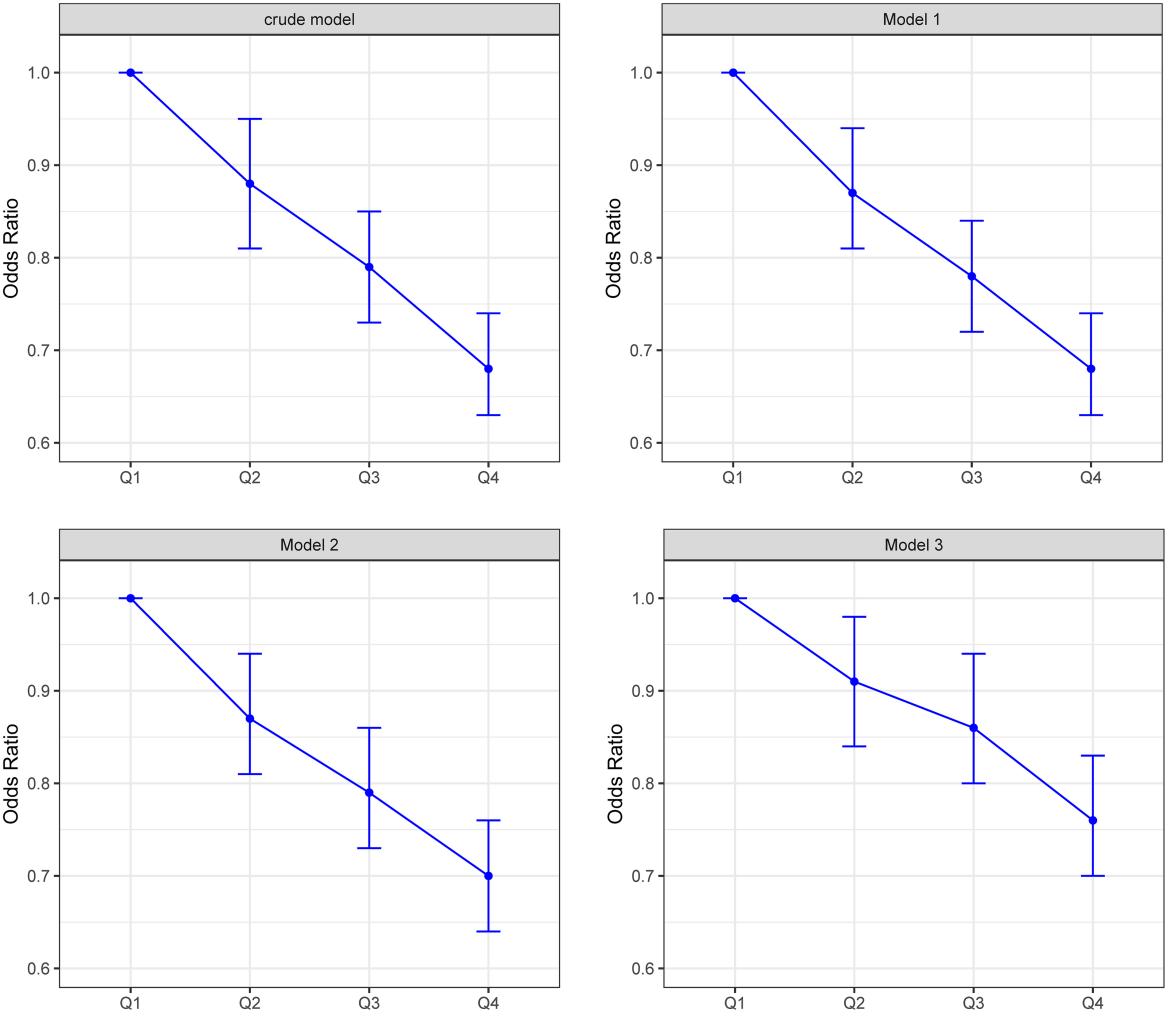
Risk of elevated depressive symptoms across HRR quantiles in four regression models.

**Figure 3:**
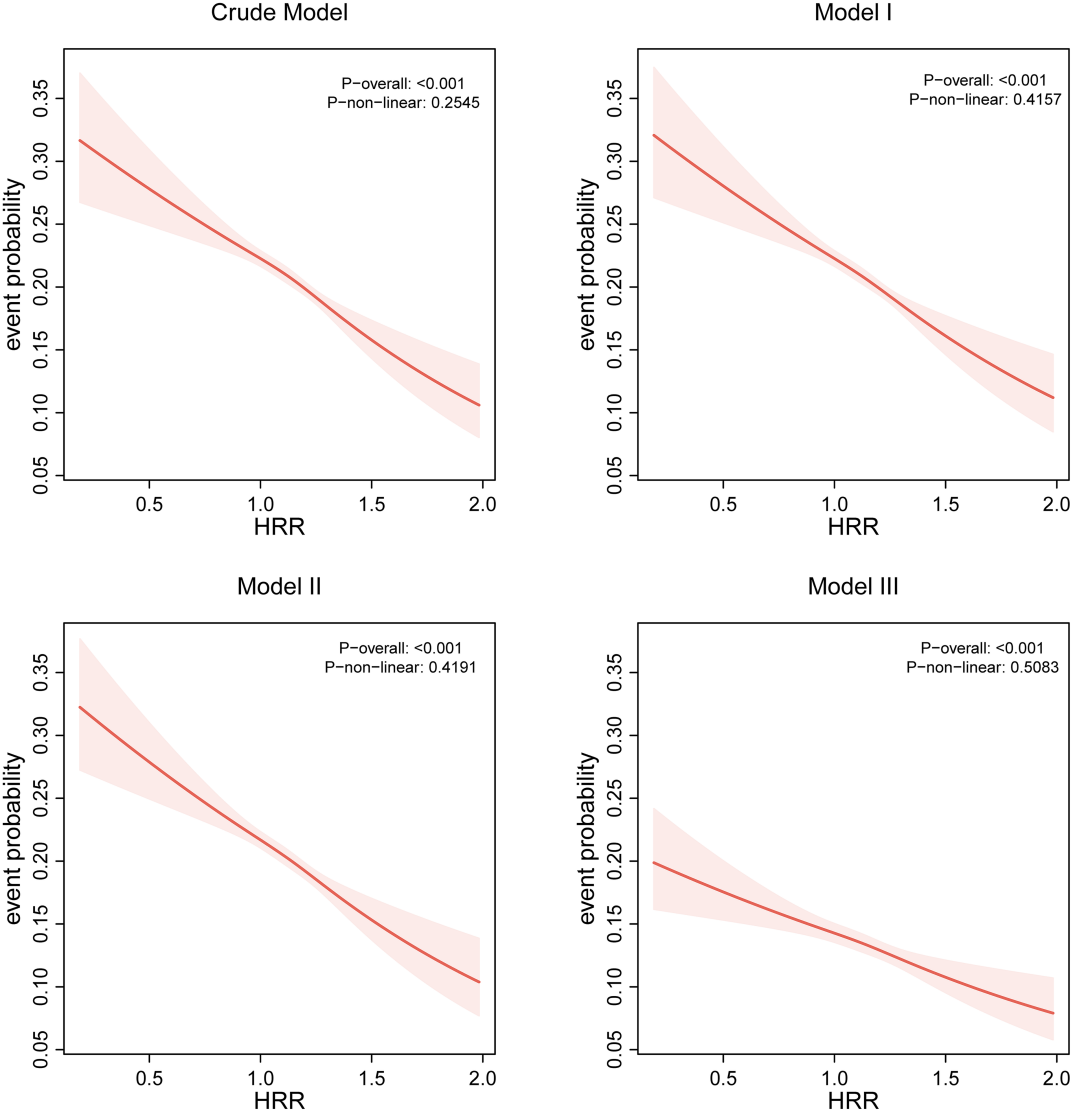
Relationship between HRR and risk of elevated depressive symptoms.

Logistic regression analyses revealed a consistent inverse graded statistical association between HRR and risk of elevated depressive symptoms across progressively adjusted models (Table 2). In unadjusted analyses, each unit elevation in HRR as a continuous variable was associated with a 52% lower risk of elevated depressive symptoms (Crude OR = 0.48, 95% CI: 0.41–0.56, *p* < 0.0001). This protective effect persisted through sequential adjustments: controlling for age and sex attenuated the association slightly (Model 1 OR = 0.49, 95% CI: 0.41–0.57, *p* < 0.0001), while further inclusion of BMI and lifestyle factors (smoking/drinking) maintained a 49% risk reduction (Model 2 OR = 0.51, 95% CI: 0.43–0.60, *p* < 0.0001). Even after comprehensive adjustment for socioeconomic (education/occupation) and cardiometabolic confounders (hypertension/diabetes/hyperlipidemia), HRR retained significant predictive capacity, with each unit increase conferring 39% lower odds of elevated depressive symptoms (Model 3 OR = 0.61, 95% CI: 0.51–0.72, *p* < 0.0001). Quartile-based analysis revealed a dose–response pattern: compared to the lowest quartile (Q1: ≤1.031), participants in higher HRR quartiles demonstrated progressively lower risks, with the most pronounced effect in Q4 (≥1.25) showing 24% risk reduction after full adjustment (OR = 0.76, 95% CI: 0.70–0.83, *p* < 0.0001). The quartile-stratified analysis demonstrated a progressive risk of elevated depressive symptoms reduction with ascending HRR levels in fully adjusted models (Table 2; Figure 2). Compared to the lowest quartile (Q1 ≤ 1.031), participants in Q2 (1.031–1.142) exhibited 10% lower risk (OR = 0.90, 95% CI: 0.84–0.98, *p* = 0.01), while those in Q3 (1.142–1.25) and Q4 (≥1.25) showed 13% (OR = 0.87, 95% CI: 0.80–0.94, *p* < 0.001) and 24% (OR = 0.76, 95% CI: 0.70–0.83, *p* < 0.0001) reduced odds, respectively. A strong dose–response pattern was confirmed by the significant trend test (*p* < 0.0001). Restricted cubic spline analyses confirmed a linear rather than threshold relationship, with risk of elevated depressive symptoms progressively decreasing across the entire HRR spectrum in all models (Figure 3). Multicollinearity diagnostics revealed acceptable levels for all covariates, with adjusted variance inflation factors ranging from 1.00 to 1.80, all below the recommended threshold of 2.24 (Supplementary Table S3).

**Table 2:** Association between HRR quantiles and elevated depressive symptoms.

HRR categories	Crude model		Model 1		Model 2		Model 3	
	OR (95%CI)	*p*-value	OR (95%CI)	*p*-value	OR (95%CI)	*p*-value	OR (95%CI)	*p*-value
HRR as continuous	0.48 (0.41,0.56)	<0.0001	0.49 (0.41,0.57)	<0.0001	0.51 (0.43,0.60)	<0.0001	0.61 (0.51,0.72)	<0.0001
HRR as quantile								
Q1 (<=1.031)	Ref		Ref		Ref		Ref	
Q2(1.031,1.142)	0.87 (0.81,0.94)	<0.001	0.87 (0.81,0.94)	<0.001	0.87 (0.80,0.94)	<0.001	0.90 (0.84,0.98)	0.01
Q3 (1.142,1.25)	0.79 (0.73,0.85)	<0.0001	0.79 (0.73,0.85)	<0.0001	0.8 (0.74,0.86)	<0.0001	0.87 (0.80,0.94)	<0.001
Q4 (> = 1.25)	0.68 (0.63,0.73)	<0.0001	0.69 (0.63,0.74)	<0.0001	0.7 (0.64,0.76)	<0.0001	0.76 (0.70,0.83)	<0.0001
*p* for trend		<0.0001		<0.0001		<0.0001		<0.0001

Model 1 adjusted for: age, sex.

Model 2 adjusted for: age, sex, BMI, smoke, drink.

Model 3 adjusted for: age, sex, bmi, smoke, drink, education, occupation, hypertension, diabetes, hyperlipidemia.

Additionally, ROC curve analysis demonstrated that HRR exhibited superior predictive performance for elevated depressive symptoms compared to individual hematological parameters. HRR achieved an AUC of 0.630 (95% CI: 0.623–0.638), significantly outperforming hemoglobin (AUC = 0.542, 95% CI: 0.534–0.550) and RDW (AUC = 0.518, 95% CI: 0.510–0.527) (both *p* < 0.001, Supplementary Figure S2). These results confirm that the composite HRR index provides enhanced discriminatory ability over conventional hematological markers for identifying individuals with elevated depressive symptoms. The discriminative ability of HRR for elevated depressive symptoms showed an AUC of 0.63, a value that falls within the range (around 0.60–0.65) generally recognized in psychiatric epidemiology as providing useful, albeit moderate, predictive information for biomarkers. This AUC value thus confirms the meaningful predictive value of HRR in the context of elevated depressive symptoms assessment and to be more clear, this level of discrimination suggests HRR may provide incremental value when combined with demographic and clinical factors, but is insufficient as a stand-alone diagnostic marker.

### Sensitivity analysis excluding anemia and kidney disease populations

To evaluate the robustness of the observed association, we conducted a sensitivity analysis excluding participants with anemia (<13 g/dL for male and <12 g/dL for female) (25) and those with kidney disease based on self-report (Supplementary Table S4). A total of 2,618 individuals with anemia and 104 with kidney disease (2,713 in total, accounting for overlapping cases) were removed from the analytic sample. The inverse association between HRR and elevated depressive symptoms remained consistent in both direction and magnitude. In the unadjusted model, each unit increase in HRR was associated with a 63% lower risk of elevated depressive symptoms (Crude OR = 0.37, 95% CI: 0.30–0.46, *p* < 0.0001), and this protective association persisted after progressive adjustment for demographic, lifestyle, socioeconomic, and cardiometabolic factors (Model 3 OR = 0.56, 95% CI: 0.45–0.70, *p* < 0.0001). Quartile analysis similarly showed a graded protective effect, with the highest quartile (Q4) having 24% lower odds of elevated depressive symptoms compared with Q1 after full adjustment (OR = 0.76, 95% CI: 0.69–0.83, *p* < 0.0001; p for trend < 0.0001) (Supplementary Table S4). These results indicate that the association between HRR and risk of elevated depressive symptoms is unlikely to be driven by participants with anemia or kidney disease.

### Sensitivity to unmeasured confounding

To quantify robustness to unmeasured confounding, we computed the E-value for the association between HRR and elevated depressive symptoms. For the highest versus lowest HRR quartile comparison (OR = 0.76), the calculated E-value was 1.88 (Supplementary Figure S1). Thus, an unmeasured confounder would need to be associated with both HRR and elevated depressive symptoms by a risk ratio of at least 1.88 each, above and beyond the measured covariates, to explain away the observed association. This indicates moderate robustness to potential unmeasured confounding.

### Subgroup and interaction analyses

Age significantly modified HRR-depression associations (*p*-interactio*n* = 0.004), with greater protection in older adults (≥45 years: OR = 0.401, 95% CI: 0.318–0.505) versus younger individuals (<45 years: OR = 0.639) (Table 3). No other subgroups showed significant interactions (all p-interaction >0.05), including sex (*p* = 0.26), BMI (*p* = 0.127), smoking (*p* = 0.898), and cardiometabolic conditions (hypertension *p* = 0.931; diabetes *p* = 0.851). Protective effects remained consistent across occupational (*p* = 0.895) and educational strata (*p* = 0.094), confirming HRR’s robust elevated depressive symptoms prevention capacity across diverse populations.

**Table 3:** Subgroup analyses and interaction tests for the association between HRR and elevated depressive symptoms risk.

Stratification factors	OR (95%CI)	*p* for interaction
Age class		0.004
< 45	0.639 (0.498,0.821)	
> = 45	0.401 (0.318,0.505)	
Sex		0.26
Male	0.463 (0.364,0.589)	
Female	0.563 (0.445,0.714)	
BMI_class		0.127
(24, 28)	0.416 (0.310,0.561)	
< 24	0.612 (0.490,0.766)	
> = 28	0.497 (0.283,0.879)	
Smoke		0.898
Never	0.518 (0.426,0.630)	
Current	0.488 (0.340,0.700)	
Past	0.570 (0.232,1.417)	
Drink		0.268
Never	0.539 (0.451,0.645)	
Current	0.363 (0.219,0.603)	
Past	0.254 (0.039,1.640)	
Occupation		0.895
Not record	0.609 (0.300,1.246)	
Freelance/Other	0.585 (0.449,0.763)	
Government/Institution	0.545 (0.407,0.732)	
Student/Retired	0.568 (0.322,1.005)	
Agriculture/Industrial	0.679 (0.396,1.167)	
Education		0.094
Not record	0.618 (0.304,1.268)	
Secondary school or vocational school	0.847 (0.627,1.145)	
College or above	0.548 (0.426,0.706)	
Elementary school or below	0.476 (0.307,0.738)	
Hypertension		0.931
No	0.521 (0.433,0.627)	
Yes	0.478 (0.314,0.730)	
Diabetes		0.851
No	0.497 (0.417,0.593)	
Yes	0.501 (0.277,0.911)	
Hyperlipidemia		0.998
No	0.512 (0.432,0.606)	
Yes	0.535 (0.125,2.391)	

Adjusted for: age, sex, BMI, smoke, drink, education, occupation, hypertension, diabetes, hyperlipidemia except the modifier.

## Discussion

In this large population-based cross-sectional study, we found a significant and linear inverse association between the hemoglobin-to-red cell distribution width ratio (HRR) and the risk of elevated depressive symptoms in Chinese adults. After full adjustment for demographic, behavioral, and clinical covariates, each unit increase in HRR was associated with a 39% lower risk of elevated depressive symptoms (OR = 0.61, 95% CI: 0.51–0.72, *p* < 0.0001). Participants in the highest HRR quartile (≥1.25) had a 24% lower risk compared to those in the lowest quartile (≤1.031) (OR = 0.76, 95% CI: 0.70–0.83). Restricted cubic spline analyses further confirmed the linearity of the association. Subgroup analysis revealed a stronger inverse relationship in individuals aged ≥45 years (OR = 0.40), with a significant age interaction (p-interactio*n* = 0.004), suggesting the enhanced value of HRR in aging populations. Importantly, this association was demonstrated in a large, real-world sample of Chinese adults undergoing routine health examinations, adding valuable population-specific evidence from an Asian context where such data remain limited.

Our findings are consistent with and extend those of a recent study by Xi, L., et al. (10), which was the first to report a significant inverse relationship between HRR and depressive symptoms in older adults (aged ≥60 years). However, their study was limited to a smaller older population (*n* = 6,926) and did not explore potential effect modifiers. By including over 30,000 adults with a broader age range (mean age ≈ 45 years) and systematically evaluating effect modification, our study confirms that the HRR-depression association persists in non-older populations and is even more pronounced among older adults within a general group (26). Additionally, we applied more comprehensive multivariable models and restricted spline analyses, providing stronger statistical evidence for the linearity and robustness of this association. Our findings establish the hemoglobin-to-red cell distribution width ratio (HRR) as a practical biomarker for risk of elevated depressive symptoms stratification, functioning as a physiological “dual-signal monitor” that captures both inflammatory stress (through RDW fluctuations) and oxygen transport efficiency (via hemoglobin levels). Unlike previous studies limited to older groups, our real-world validation across working-age adults demonstrates HRR’s particular utility in primary care screening settings where rapid, cost-effective tools are needed—though implementation requires addressing device standardization challenges. Looking forward, translating this discovery into clinical impact necessitates: nutritional intervention trials targeting HRR optimization, longitudinal validation in diverse populations, and development of automated analysis systems integrating HRR calculation into routine blood test workflows.

Recent literature has also explored other inflammation-related hematological ratios in relation to elevated depressive symptoms. For instance, studies have examined the RDW-to-albumin ratio (RAR), another composite biomarker reflecting inflammation and nutritional status (27). A study by Liu et al. (8) reported that elevated RAR was significantly associated with increased risk of depressive symptoms in middle-aged and older adults in the NHANES population. Similarly, Zhou et al. (7) confirmed a positive linear association between RAR and risk of elevated depressive symptoms using restricted cubic splines. While both RAR and HRR integrate RDW, our study uniquely investigates HRR, which incorporates hemoglobin—a marker directly related to oxygen transport and energy metabolism (28). In contrast to the positive correlation seen with RAR, our findings suggest that HRR has a negative association with risk of elevated depressive symptoms, reinforcing the hypothesis that both elevated RDW and lower Hb levels contribute independently and synergistically to elevated depressive symptoms vulnerability. The divergence in direction between RAR and HRR also underscores the importance of the numerator in these ratios—albumin as a nutritional marker versus hemoglobin as a functional metabolic indicator (29). Meanwhile, to assess how strong an unmeasured confounder would have to be to negate an observed association, we use the E-value to offer an interpretable metric. In our study, an E-value of 1.88 suggests that only a relatively strong unmeasured factor—linked to both HRR and elevated depressive symptoms by risk ratios approaching ~1.9—could fully explain away the observed association. Although E-values do not eliminate the possibility of residual confounding, they contextualize credibility in observational research and complement multivariable adjustment and subgroup/sensitivity analyses. Clinically, this degree of robustness supports the premise that HRR merits further evaluation as a pragmatic screening biomarker, while prospective studies with richer covariate measurement remain necessary.

Our study transforms a routine blood test into a elevated depressive symptoms detection tool through several innovations. First, we validated the hemoglobin-RDW ratio (HRR) in over 30,000 diverse adults at West China Hospital - capturing real-world complexity often missed in controlled trials (11). Second, our multidimensional analysis revealed how age fine-tunes HRR’s predictive power, allowing tailored screening approaches across life stages (30). Most importantly, HRR’s magic lies in its clinical practicality: derived from standard CBC tests costing less than a coffee, it delivers insights previously requiring expensive neuroimaging. What’s more, unlike other biomarkers, HRR uniquely combines two biological narratives: red blood cell stability and oxygen delivery efficiency (29). This dual perspective helps explain why it outperforms alternatives like RAR in detecting elevated depressive symptom’s physical roots (31, 32). Last but not least, methodologically, our work differs from secondary analyses of open repositories (e.g., NHANES) that have recently raised concerns regarding oversimplified univariate designs and data dredging: we used a single-center clinical dataset with standardized, in-person symptom assessment and unified laboratory protocols, prespecified inclusion/exclusion criteria, and exclusively multivariable modeling with transparent reporting (33, 34). These features reduce several risks highlighted by recent editorials and guidance for analyses of large health datasets.

Nonetheless, limitations remain. Firstly, the exclusion of cardiovascular disease and cancer cases enhances causal inference validity but restricts immediate applicability to clinical populations. Our findings primarily inform risk of elevated depressive symptoms stratification in community-dwelling Chinese adults without these conditions. Secondly, because exposure and outcome were measured concurrently, reverse causation cannot be excluded; prospective cohort studies are required to establish temporal sequence. Thirdly, while SDS use aligns with China’s health screening protocols (3), we acknowledge potential comparability limitations with newer instruments like PHQ-9. And future studies should implement dual-assessment designs to evaluate HRR’s performance across diagnostic standards. What’s more, despite adjustment for a range of covariates (age, sex, BMI, smoking, alcohol, education, occupation, hypertension, diabetes, hyperlipidemia, and self-reported kidney disease), we were unable to measure important potential confounders — notably nutritional status/iron indices (ferritin, transferrin saturation), systemic inflammatory markers (CRP, IL-6), detailed medication histories (including antidepressants and anti-inflammatory drugs), and psychosocial stressors. These unmeasured factors could partially account for the observed associations (35–37). Lastly, the potential overlap between HRR and comorbid anemia or chronic disease states should be interpreted with caution.

Despite these limitations, our findings highlight the potential role of HRR in elevated depressive symptoms research. Firstly, it is worth noting that reverse causation is plausible; psychological symptoms associated with depression (reduced appetite, poor self-care, altered sleep) may lead to nutritional deficiencies and systemic inflammation, which in turn can lower hemoglobin and increase RDW. Therefore, observed associations could reflect the biological consequences of depressive symptoms rather than etiologic antecedents. Secondly, we use ROC curve analysis demonstrated that HRR achieved an AUC of 0.630 (95% CI: 0.623–0.638) and falls within a range often regarded as meaningful in psychiatric epidemiology. Given the modest AUC, HRR should not be used as a stand-alone classifier; rather, it may serve as a supplementary marker to improve multivariable risk stratification pending prospective validation. Importantly, HRR is inexpensive, routinely measured in clinical practice, biologically plausible given the interplay between hemoglobin, RDW, and systemic inflammation, and easily reproducible across populations. Therefore, while HRR alone should not be considered a definitive diagnostic marker for elevated depressive symptoms, it may provide incremental value when integrated with conventional demographic and clinical risk factors.

Future longitudinal and multi-center studies are warranted to further validate its predictive performance and to explore its potential utility in early screening, individualized prevention, and risk stratification strategies in both clinical and public health contexts. What’s more, future studies could employ longitudinal designs and, where appropriate, advanced causal inference techniques — for example, propensity score matching or inverse probability weighting to reduce measured confounding, instrumental variable analyses or Mendelian randomization to probe causality, and mediation analyses incorporating measured inflammatory and iron biomarkers to disentangle biological pathways.

### Conclusion

In this large cross-sectional sample of Chinese adults, higher HRR was associated with lower prevalence of elevated depressive symptoms, particularly among individuals aged ≥45 years. These findings suggest HRR may serve as a readily available biomarker for elevated depressive symptoms screening in clinical practice. However, these results are preliminary and hypothesis-generating; prospective studies measuring additional biological and psychosocial covariates are required to determine causality and clinical utility.

## Data Availability

The original contributions presented in the study are included in the article/Supplementary material, further inquiries can be directed to the corresponding author/s.
