# Supplementary material for: Association between hemoglobin-to-red cell distribution width ratio and depression in Chinese adults: a population-based cross-sectional study
Source: Front Public Health. 2025 Sep 16;13:1638290. doi: 10.3389/fpubh.2025.1638290 (PMC12479292; doi:10.3389/fpubh.2025.1638290)
Supplement: Supplementary file 1 [file Supplementary_file_1.docx]

***Supplementary Material***

**Association Between Hemoglobin-to-Red Cell Distribution Width Ratio and Depression in Chinese Adults: A Population-Based Cross-Sectional Study**

Yike Xu^1#^ , Shuwen Zhang^2,3#^ , Yang Liu^4,5^ , Junteng Zhou^6*^

^1^West China School of Medicine, West China Hospital, Sichuan University, Chengdu, 610041, China

^2^Department of Cardiology and Laboratory of Cardiovascular Diseases, West China Hospital, Institute of Cardiovascular Diseases, Sichuan University, Chengdu, Sichuan 610041, China

^3^Regenerative Medicine Research Center, West China Hospital, Sichuan University, Chengdu 610041, China.

^4^Key Laboratory of Birth Defects and Related Diseases of Women and Children of MOE, State Key Laboratory of Biotherapy, West China Second University Hospital, Sichuan University, No.17 People's South Road, Chengdu, Sichuan 610041, China.

^5^Division of Vascular Surgery, Department of General Surgery, and Laboratory of Cardiovascular Diseases, West China Hospital, Sichuan University, No.17 People's South Road, Chengdu, Sichuan 610041, China.

^6^Health Management Center, General Practice Medical Center, West China Hospital, Sichuan University, Chengdu 610041, China

*Correspondence to: Junteng Zhou, Health Management Center, General Practice Medical Center, West China Hospital, Sichuan University, Chengdu 610041, China

E-mail: [zhoujunteng@scu.edu.cn](mailto:zhoujunteng@scu.edu.cn)

# Yike Xu and Shuwen Zhang contributed equally to this work.

Table S1. Characteristics of the participants excluded from the analyses and those included in the final analyses.

|  | **Total (n=63564)** | **Excluded  (n=33137)** | **Included  (n=30427)** |
| --- | --- | --- | --- |
| **Age, years** | 46.41 ± 12.03 | 47.79 ± 12.96 | 44.91 ± 10.72 |
| **Sex** |  |  |  |
| Female | 29799(46.88) | 15717(47.43) | 14082(46.28) |
| Male | 33765(53.12) | 17420(52.57) | 16345(53.72) |
| **BMI** | 23.65 ± 3.48 | 23.57 ± 3.35 | 23.73 ± 3.59 |
| **Smoke** |  |  |  |
| Current | 12106(19.05) | 5731(17.29) | 6375(20.95) |
| Never | 48809(76.79) | 26009(78.49) | 22800(74.93) |
| Past | 2649( 4.17) | 1397( 4.22) | 1252( 4.11) |
| **Drink** |  |  |  |
| Current | 6137( 9.65) | 2702( 8.15) | 3435(11.29) |
| Never | 56910(89.53) | 30156(91.00) | 26754(87.93) |
| Past | 517( 0.81) | 279( 0.84) | 238( 0.78) |
| **Occupation** |  |  |  |
| Agriculture/Industrial | 4223( 6.64) | 1974( 5.96) | 2249( 7.39) |
| Freelance/Other | 18626(29.30) | 7547(22.78) | 11079(36.41) |
| Government/Institution | 30023(47.23) | 17557(52.98) | 12466(40.97) |
| Not record | 3233( 5.09) | 1631( 4.92) | 1602( 5.27) |
| Student/Retired | 7459(11.73) | 4428(13.36) | 3031( 9.96) |
| **Education** |  |  |  |
| College or above | 38286(60.23) | 21025(63.45) | 17261(56.73) |
| Elementary school or below | 6822(10.73) | 3344(10.09) | 3478(11.43) |
| Not record | 3184( 5.01) | 1615( 4.87) | 1569( 5.16) |
| Secondary school or vocational school | 15272(24.03) | 7153(21.59) | 8119(26.68) |
| **Hypertension** |  |  |  |
| No | 52013(81.83) | 26792(80.85) | 25221(82.89) |
| Yes | 11551(18.17) | 6345(19.15) | 5206(17.11) |
| **Diabetes** |  |  |  |
| No | 58446(91.95) | 30432(91.84) | 28014(92.07) |
| Yes | 5118( 8.05) | 2705( 8.16) | 2413( 7.93) |
| **Hyperlipidemia** |  |  |  |
| No | 62522(98.36) | 32589(98.35) | 29933(98.38) |
| Yes | 1042( 1.64) | 548( 1.65) | 494( 1.62) |
| **RDW (%)** | 13.19 ± 1.14 | 13.20 ± 1.13 | 13.18 ± 1.15 |
| **HB (g/dL)** | 147.30 ± 16.46 | 146.83 ± 16.20 | 147.76 ± 16.71 |
|  |  |  |  |

Table S2. Baseline characteristics of participants by HRR quantiles.

|  | Q1(<=1.031) (n=7559) | Q2(1.031,1.142) (n=7663) | Q3(1.142,1.25)  (n=7705) | Q4 (>=1.25) (n=7500) | p-value |
| --- | --- | --- | --- | --- | --- |
| Age | 45.44 ± 10.64 | 45.75 ± 11.10 | 45.38 ± 10.50 | 43.02 ± 10.38 | <0.0001 |
| Sex |  |  |  |  | 0.57 |
| Female | 3538(46.81) | 3567(46.55) | 3536(45.89) | 3441(45.88) |  |
| Male | 4021(53.19) | 4096(53.45) | 4169(54.11) | 4059(54.12) |  |
| BMI, kg/m2 | 22.50 ± 3.20 | 22.99 ± 3.33 | 24.32 ± 3.58 | 25.13 ± 3.63 | <0.0001 |
| Smoke |  |  |  |  | 0.06 |
| Current | 1577(20.86) | 1683(21.96) | 1581(20.52) | 1534(20.45) |  |
| Never | 5656(74.82) | 5660(73.86) | 5840(75.79) | 5644(75.25) |  |
| Past | 326( 4.31) | 320( 4.18) | 284( 3.69) | 322( 4.29) |  |
| Drink |  |  |  |  | 0.99 |
| Current | 858(11.35) | 854(11.14) | 858(11.14) | 865(11.53) |  |
| Never | 6642(87.87) | 6748(88.06) | 6786(88.07) | 6578(87.71) |  |
| Past | 59( 0.78) | 61( 0.80) | 61( 0.79) | 57( 0.76) |  |
| Occupation |  |  |  |  | <0.0001 |
| Agriculture/Industrial | 693( 9.17) | 552( 7.20) | 532( 6.90) | 472( 6.29) |  |
| Freelance/Other | 3008(39.79) | 2815(36.73) | 2660(34.52) | 2596(34.61) |  |
| Government/Institution | 2543(33.64) | 2875(37.52) | 3437(44.61) | 3611(48.15) |  |
| Not record | 429( 5.68) | 382( 4.98) | 402( 5.22) | 389( 5.19) |  |
| Student/Retired | 886(11.72) | 1039(13.56) | 674( 8.75) | 432( 5.76) |  |
| Education |  |  |  |  | <0.0001 |
| College or above | 3772(49.90) | 4191(54.69) | 4559(59.17) | 4739(63.19) |  |
| Elementary school or below | 986(13.04) | 895(11.68) | 851(11.04) | 746( 9.95) |  |
| Not record | 422( 5.58) | 368( 4.80) | 395( 5.13) | 384( 5.12) |  |
| Secondary school or vocational school | 2379(31.47) | 2209(28.83) | 1900(24.66) | 1631(21.75) |  |
| Hypertension |  |  |  |  | <0.0001 |
| No | 6555(86.72) | 6506(84.90) | 6214(80.65) | 5946(79.28) |  |
| Yes | 1004(13.28) | 1157(15.10) | 1491(19.35) | 1554(20.72) |  |
| Diabetes |  |  |  |  | <0.0001 |
| No | 7202(95.28) | 7155(93.37) | 6984(90.64) | 6673(88.97) |  |
| Yes | 357( 4.72) | 508( 6.63) | 721( 9.36) | 827(11.03) |  |
| Hyperlipidemia |  |  |  |  | <0.0001 |
| No | 7497(99.18) | 7579(98.90) | 7543(97.90) | 7314(97.52) |  |
| Yes | 62( 0.82) | 84( 1.10) | 162( 2.10) | 186( 2.48) |  |
| Kidney disease |  |  |  |  | 0.28 |
| No | 7538(99.72) | 7635(99.63) | 7683(99.71) | 7467(99.56) |  |
| Yes | 21( 0.28) | 28( 0.37) | 22( 0.29) | 33( 0.44) |  |
| Hb (g/dL) | 12.84 ± 1.14 | 14.22 ± 0.78 | 15.40 ± 0.78 | 16.65 ± 0.87 | <0.0001 |
| RDW (%) | 14.27 ± 1.60 | 13.06 ± 0.65 | 12.88 ± 0.59 | 12.49 ± 0.51 | <0.0001 |
| HRR | 0.91 ± 0.12 | 1.09 ± 0.03 | 1.20 ± 0.03 | 1.33 ± 0.07 | <0.0001 |
| Depression (%) |  |  |  |  | <0.0001 |
| No | 5762(76.23) | 6026(78.64) | 6186(80.29) | 6192(82.56) |  |
| Yes | 1797(23.77) | 1637(21.36) | 1519(19.71) | 1308(17.44) |  |

HRR: hemoglobin-to-red blood cell distribution width ratio

| Table S3. Multicollinearity diagnostics for the fully adjusted regression models | | | | | |
| --- | --- | --- | --- | --- | --- |
|  |  |  |  |  |  |
| Variable | HRR Continuous Model | |  | HRRQ Categorical Model | |
|  | GVIF | GVIF^(1/(2*Df)) |  | GVIF | GVIF^(1/(2*Df)) |
| HRR/HRRQ | 1.11 | 1.05 |  | 1.15 | 1.02 |
| Age | 1.4 | 1.18 |  | 1.41 | 1.19 |
| Sex | 1.13 | 1.06 |  | 1.13 | 1.06 |
| BMI | 1.21 | 1.1 |  | 1.23 | 1.11 |
| Smoking status | 1 | 1 |  | 1 | 1 |
| Drinking status | 1.13 | 1.03 |  | 1.13 | 1.03 |
| Education | 34.08 | 1.8 |  | 34.13 | 1.8 |
| Occupation | 33.41 | 1.55 |  | 33.52 | 1.55 |
| Hypertension | 1.18 | 1.09 |  | 1.18 | 1.09 |
| Diabetes | 1.09 | 1.04 |  | 1.09 | 1.04 |
| Hyperlipidemia | 1.02 | 1.01 |  | 1.02 | 1.01 |

Table S4 Association between HRR quantiles and risk of elevated depressive symptoms in populations without anemia and kidney disease

|  |  |  |  |  |  |  |  |  |
| --- | --- | --- | --- | --- | --- | --- | --- | --- |
|  | crude model |  | Model 1 |  | Model 2 |  | Model 3 |  |
|  | OR (95%CI) | p-value | OR (95%CI) | p-value | OR (95%CI) | p-value | OR (95%CI) | p-value |
| HRR as continuous | 0.37(0.30,0.46) | <0.0001 | 0.43(0.35,0.53) | <0.0001 | 0.46(0.37,0.57) | <0.0001 | 0.56(0.45,0.70) | <0.0001 |
| HRR as quantile |  |  |  |  |  |  |  |  |
| Q1 (<=1.031) | ref |  | ref |  | ref |  | ref |  |
| Q2(1.031,1.142) | 0.83(0.76,0.90) | <0.0001 | 0.87(0.80,0.94) | <0.001 | 0.87(0.80,0.95) | 0.001 | 0.9(0.82,0.98) | 0.01 |
| Q3 (1.142,1.25) | 0.75(0.69,0.82) | <0.0001 | 0.79(0.72,0.86) | <0.0001 | 0.8(0.73,0.87) | <0.0001 | 0.86(0.79,0.94) | 0.001 |
| Q4 (>=1.25) | 0.64(0.59,0.70) | <0.0001 | 0.68(0.63,0.75) | <0.0001 | 0.7(0.64,0.77) | <0.0001 | 0.76(0.69,0.83) | <0.0001 |
| p for trend |  | <0.0001 |  | <0.0001 |  | <0.0001 |  | <0.0001 |

Model 1 adjusted for : Age, Sex

Model 2 adjusted for : Age, Sex, BMI, Smoke, Drink

Model 3 adjusted for : Age, Sex, BMI, Smoke, Drink, Education, Occupation, Hypertension, Diabetes, Hyperlipidemia


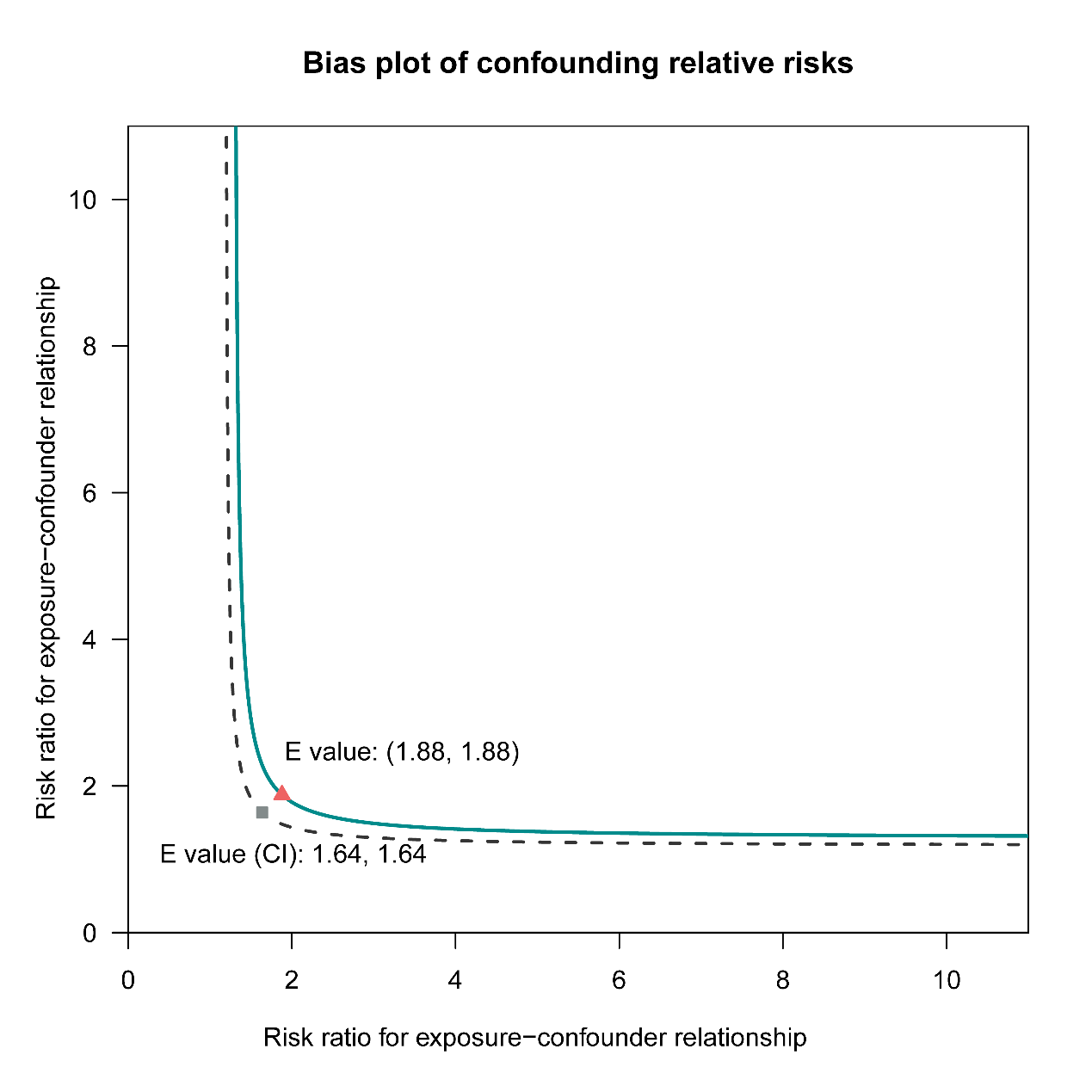


Figure S1. Value of the joint minimum strength of association on the risk ratio scale that an unmeasured confounder would be required to have with plasma HRR, and depression risk (the outcome).


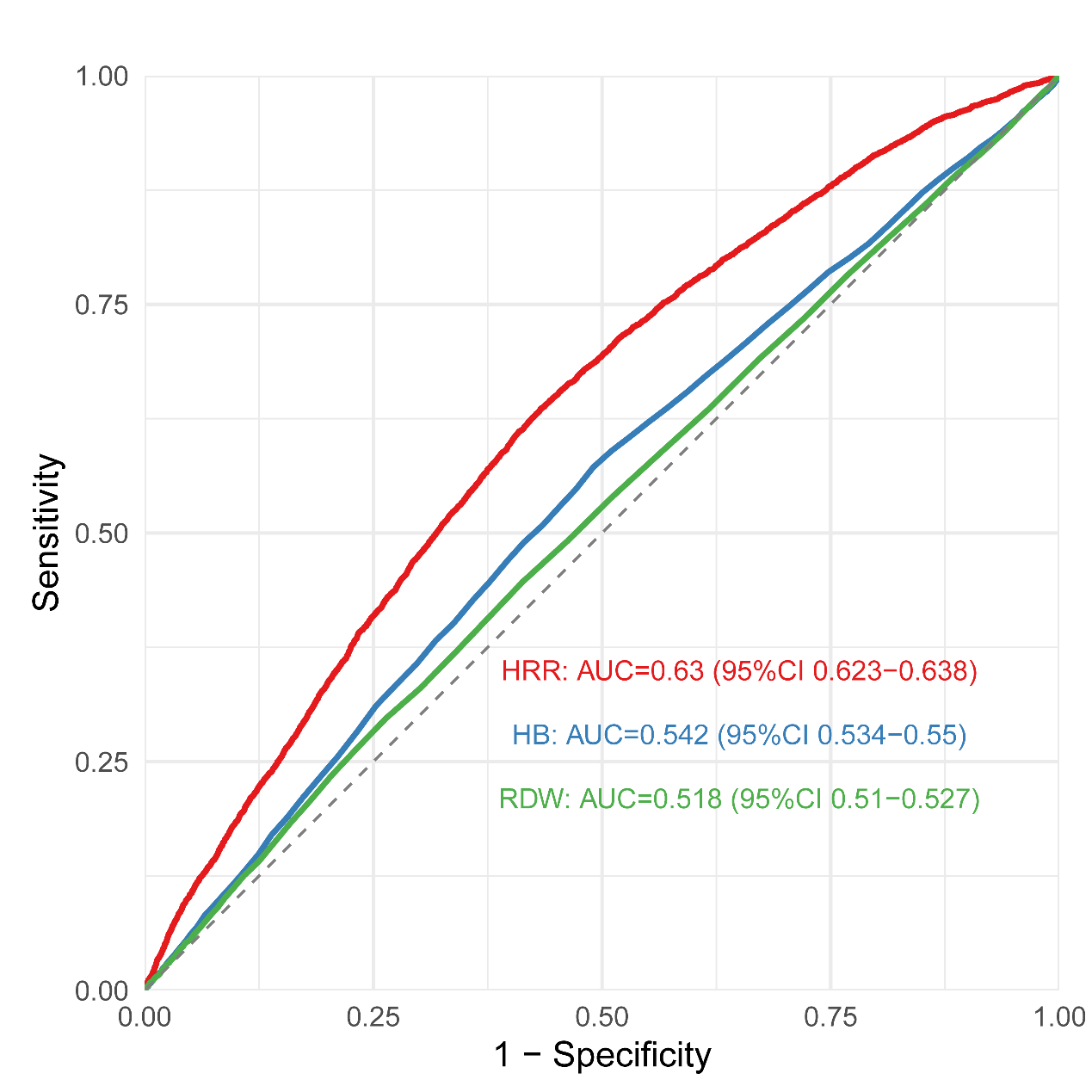


Figure S2. ROC Curves for Depression Prediction
